# An improved diagrammatic procedure for interpreting and scoring the Wisconsin Card Sorting Test: An update to Steve Berry’s 1996 edition

**DOI:** 10.3758/s13428-024-02499-w

**Published:** 2024-10-21

**Authors:** Caitlin A. Howlett, G. Lorimer Moseley

**Affiliations:** Innovation, Implementation & Clinical Translation (IIMPACT) in Health, University of South Australia, Kaurna Country, GPO Box 2471, Adelaide, South Australia 5001 Australia

**Keywords:** Executive function, WCST, Scoring, Interpretation, Neuropsychology, Neuropsychological assessment, Decision tree

## Abstract

The Wisconsin Card Sorting Test (WCST) is a popular neuropsychological test that is complicated to score and interpret. In an attempt to make scoring of the WCST simpler, Berry (The Clinical Neuropsychologist 10, 117–121, 1996) developed a diagrammatic scoring procedure, particularly to aid scoring of perseverative responses. We identified key limitations of Berry’s diagram, including its unnecessary ambiguity and complexity, use of terminology different from that used in the standardized WCST manual, and lack of distinction between perseverative errors and perseverative responses. Our new diagrammatic scoring procedure scores each response one-by-one; we strongly suggest that the diagram is used in conjunction with the 1993 WCST manual. Our new diagrammatic scoring procedure aims to assist novice users in learning how to accurately score the task, prevent scoring errors when using the manual version of the task, and help scorers verify whether other existing computerized versions of the task (apart from the PAR version) conform to the Heaton et al. (1993) scoring method. Our diagrammatic scoring procedure holds promise to be incorporated into any future versions of the WCST manual.

## The historical context of the WCST

The Wisconsin Card Sorting Test (WCST) is a well-known neuropsychological test that has been widely used to assess various domains of executive function (Rabin et al., 2005). Two key outcome variables derived from the WCST include what are termed *perseverative responses* and *perseverative errors* (Heaton et al., 1993). Scoring perseveration on the WCST has long been recognized as a rather complicated process (Axelrod et al., 1992; Greve, 1993; Miles et al., 2021). In the early 1990s, researchers attempted to simplify and clarify the rules for scoring perseverative responses and perseverative errors by creating supplementary scoring materials (Axelrod et al., 1992; Flashman et al., 1991), which were intended to be used in conjunction with the 1981 WCST standardized manual (Heaton, 1981). Published in 1993, the updated version of the WCST manual (Heaton et al., 1993) incorporated the suggestions made by Axelrod et al. (1992) and Flashman et al. (1991). In recent years, attempts have been made to shed light on the lack of consensus surrounding the key terminology, definitions, and scoring methods of the WCST, and clarify key variables of the task (Miles et al., 2021). It is recommended that users who are unfamiliar with the WCST refer to the Heaton et al. (1993) manual and Miles et al. (2021) for key terminology and definitions that are aligned with the contemporary version of the task.

## What makes the WCST such a complex task?

Several issues make the WCST very complex to score and interpret. First, the principle that determines whether a response is scored as perseverative or not – the ‘perseverated-to principle’ – is dictated by the examinee’s responses (Berry, 1996). This means that the perseverated-to principle can change throughout the task and is not simply the previous sorting rule, which is a common misunderstanding that is evident in the scientific literature. Second, each response is evaluated along three scoring dimensions – correct or incorrect, ambiguous or unambiguous, and perseverative or non-perseverative. That is, participant responses on the WCST cannot be scored in isolation but must be considered in the context of all other previous and subsequent responses. For instance, for an ambiguous response to be scored as a perseverative response, it needs to be ‘sandwiched’ between two unambiguous perseverative errors and must match the perseverated-to principle that is currently in effect. Therefore, the WCST should only be scored once the examinee has completed the task. Indeed, previous research has found that failing to detect a change to the perseverated-to principle (either prior to the completion of the first category i.e., color or when a sequence of three unambiguous errors is separated by ambiguous responses), and not adhering to the ‘sandwich rule’, are common mistakes among novice scorers of the WCST (Axelrod et al., 1994). Third, the distinction between perseverative responses and perseverative errors has long been a source of confusion (Axelrod et al., 1992; Greve, 1993). The key difference between the two variables is that perseverative responses (repeated card sorts) can be correct, but perseverative errors are, by definition, not. While perseverative responses are – for the most part – incorrect responses that do not match the correct sorting rule and are termed perseverative errors, perseverative responses can also be correct if the response is ambiguous (i.e., the response matches two or more dimensions, for instance, color and number). It should be noted, however, that the distinction between perseverative errors and perseverative responses is arguably of uncertain significance because the two scores are highly correlated (Bowden et al., 1998). Further, the clinical usefulness of one variable over the other remains to be established. However, some clinicians may prefer to focus on perseverative errors in lieu of perseverative responses due to the perceived inappropriateness of counting *correct responses* (i.e., perseverative tendencies) as indicative of a problem among clinical presentations.

## Prior use of decision tree diagrams to aid scoring of the WCST

The revised version of the WCST manual (Heaton et al., 1993) presents the procedures for scoring perseverative responses and perseverative errors using detailed descriptions, which are supported by illustrative examples. However, presenting the scoring procedures in this manner is time-consuming for novice users (Axelrod et al., 1992). A published version of the procedures for scoring perseverative responses in the form of diagrammatic decision trees has been previously attempted by Berry (1996); however, despite such efforts, a gap in the literature remains for a graphic representation of how to score perseveration on the WCST – one that is simple, precise, and uses terminology that aligns with the 1993 Heaton (1993) manual. A clear limitation of Berry’s (1996) diagram is that it does not differentiate between perseverative errors and perseverative responses.

**Fig. 1:**
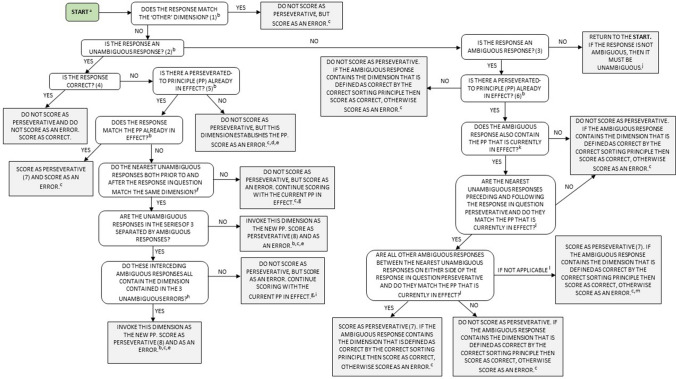
Adaptation of Steve Berry’s 1996 diagrammatic procedure for scoring perseverative responses (and perseverative errors) on the Wisconsin Card Sorting Test

We have made modifications to the original diagrams in order to overcome this limitation (greater detail is provided in Fig. 1). We have:

i) Combined Berry’s (1996) three diagrams into one (see Fig. 1) to allow users to better see how these three schematic representations are interconnected.
ii) Removed redundant information to reduce unnecessary ambiguity and complexity.
iii) Used terminology that is consistent with the updated standardized manual (e.g., replacing *“perseveration principle”* with *“perseverated-to principle”*).
iv) Included directions on how and when to score perseverative errors.
v) Included differentiation of a response as ‘correct’ or as an ‘error’.
vi) Included necessary and comprehensive notes for additional information without the need to re-locate these key points within the main text of the 234-page WCST manual. The numbers in the diagrammatic decision tree, which correspond to the referenced numbers in the notes section, should be used in conjunction with the information included in the scoring diagram to ensure accuracy and completeness.

## So, where to next?

The last comprehensive revision of the standardized WCST manual was in 1993 (Heaton et al., 1993); perhaps the field may benefit from another revision to the WCST manual so as to simplify the scoring procedures. With such complex scoring procedures, the potential for human errors is difficult to ignore. Despite computerized versions of the task being available (i.e., PAR version), accessing (> $1,000 USD) them may be difficult in low resource contexts. In such contexts, researchers and clinicians may be required to score the WCST manually. To avoid errors when scoring the task by hand, Heaton et al. may consider incorporating schematic ‘decision trees’ as part of the WCST manual. We expect this to assist users to accurately score complex responses (e.g., how to score the task when the perseverated-to principle changes, or how to score ambiguous responses). Such revisions to the WCST manual will not only improve accuracy but might also allow scorers of the task – particularly novices – to learn how to score perseverative responses and perseverative errors with more ease. It is also anticipated that such a graphical depiction of how to accurately score the WCST would prove useful to those who rely on other computerized forms of the task [apart from the PAR version (PAR staff., 2020)] as a way of verifying whether the scoring conforms to the Heaton et al. (1993) manual and reducing the chance of misinterpreting the rules in the instruction manual.

## Overarching instructions for how to use the scoring decision tree

It should be noted that this diagrammatic scoring procedure should not replace the WCST manual (1993); users are strongly encouraged to become familiar with the WCST manual before using the test. Further, our diagrammatic scoring procedure is intended to mirror the procedures for scoring perseverative responses, perseverative errors, non-perseverative errors, and total number correct, as set out by Heaton et al. (1993), and only focuses on these few variables. Therefore, the standardized manual should be referred to for information concerning the administration of the WCST, or how to score other key variables of the task. The diagram scores each response one-by-one, beginning with the very first response and involves responding to a series of yes/no questions until a grey shaded box is reached, which will outline how to score any given response. However, the WCST should only be scored at the completion of the task, rather than as the participant responds. It is also important to note that the PAR computerized version of the WCST can be used for test administration and scoring; the scoring component follows the standardized procedures specified in the Heaton et al. (1993) manual and can be used to score the WCST task more efficiently and accurately irrespective of which version of the task (i.e., manual or computerized) is administered. Use of this program can therefore avoid the potential for scoring errors and should be used where possible.

## Conclusion

The WCST is a well-known neuropsychological test that is very complicated to score and interpret (Heaton et al., 1993). We have presented a new diagrammatic scoring procedure for the WCST, having identified and resolved some important limitations of the current diagram – originally developed by Berry (1996) – particularly with regard to the distinction between perseverative responses and perseverative errors. We have modified the terminology within the diagram to better align with the 1993 standardized manual. We have found that the diagram eliminates unnecessary ambiguity, minimizes confusion for scorers of the task, and improves the instrument’s usability. Independent appraisal of our diagrammatic scoring procedure would be welcomed.

Corresponding notes^b,n^ (the numbers correspond to the footnotes presented in Fig. 1):

1. A response that does not match either color, form, or number (i.e., C F N Ø).
2. An unambiguous response is one in which the response card matches a stimulus card on only one dimension. For example, Ȼ F N O.
3. An ambiguous response is one in which the response card matches a stimulus card on more than one dimension. For example, Ȼ  N O. By definition, responses that match the ‘Other’ dimension are ambiguous.
4. A response is deemed to be *correct* when it matches the correct sorting principle that is currently in effect. If the response does not match the correct sorting principle that is currently in effect, the response is deemed to be incorrect and is scored as an *error*.
5. The perseverated-to principle (PP) is the rule that determines whether a response is considered *perseverative* or not. The PP is established in the first category (i.e., color category) once the first unambiguous error is made. The rule is determined by the examinee’s responses and as depicted in the diagram, the PP can change throughout the task:
  i. immediately after a category is completed (i.e., the response after the 10^th^ correct response), the PP is deemed to have lapsed. The PP becomes the dimension that was the correct sorting principle in the previous category
  ii. when three consecutive unambiguous errors are made in a row to a dimension that is not correct or perseverative, or
  iii. where there are ambiguous responses between these three unambiguous errors, they all match the same dimension that is neither correct, nor perseverative.
6. If an ambiguous response is made immediately after a category has been completed (i.e., occurs after 10 consecutive correct responses), it cannot be scored as perseverative.
7.  Perseverative errors are perseverative responses; however, not all perseverative responses are perseverative errors. Perseverative responses can either be correct or incorrect as a result of ambiguous responses and the ‘sandwich rule’ (i.e., an ambiguous response that is situated (‘sandwiched’) between two unambiguous perseverative errors).
8.  When new PP’s are invoked, they do not come into effect, nor are they scored as perseverative until the second unambiguous error.

Figure 1 has been adapted from Berry’s (1996) three original diagrams, which have now been combined into a single diagram. A detailed summary of changes is provided below; the superscript letters correspond to the footnotes presented in Fig. 1 (except for ‘n’) to indicate where modifications have been made:

^a^ ‘Start’ has been added to the diagram to indicate where users of the WCST should begin when scoring each individual response.

^b^ Rephrased to use terminology that is consistent with the Heaton et al. (1993) WCST manual.

^c^ Instructions for how to score perseverative and non-perseverative errors have been incorporated into the diagram.

^d^ This section of Berry’s diagram was technically inaccurate. That is, if there is no PP already in effect, then the PP is not *new* (as Berry described), but is *established*. This section has been re-worded to accurately reflect that scenario.

^e^ This section of Berry’s diagram does not allow users of the WCST to score each response on an individual basis, so this has been adapted accordingly.

^f^ ‘Closest’ has been replaced with ‘nearest’ for consistency.

^g^ ‘Previous PP’ has been replaced with ‘current PP in effect’. Using ‘previous PP’ implies that an earlier PP should be used, when in fact, the current PP remains in effect.

^h^ This section of Berry’s diagram is technically inaccurate. ‘Unambiguous responses’ has been replaced with ‘unambiguous errors’ because such responses also need to be incorrect (i.e., not matching the correct sorting principle).

^i^ Explicit instructions have been provided for how to score this scenario because Berry (1996) did not provide such instructions (i.e., whether the response should be scored as perseverative or not).

^j^ ‘See decision tree number 1’ has been replaced with ‘return to the start’ and a brief description as to why users should return to the start of the diagram has been provided.

^k^ This section has been simplified. Berry’s original phrasing included both the terms ‘category’ and ‘PP’, but introducing another term here is unnecessary.

^l^ This section has been made clearer by stipulating that the unambiguous responses preceding and following the response in question must be i) perseverative and ii) match the PP that is currently in effect.

^m^ This section has been added to the diagram so that in cases where no more than one ambiguous response is given, users of the WCST know how to score such scenarios (i.e., if not applicable, then score as perseverative).

^n^ A more comprehensive notes section has been added that provides key information to consider when scoring perseverative responses and perseverative errors.

## Data Availability

Not applicable.
